# Chronic necrotizing pulmonary aspergillosis presenting as bilateral pleural effusion: a case report

**DOI:** 10.1186/1752-1947-6-62

**Published:** 2012-02-14

**Authors:** Sakthiswary Rajalingham, Fauzi M Anshar

**Affiliations:** 1 Department of Medicine, Universiti Kebangsaan Malaysia Medical Centre, Jalan Yaacob Latif, 56000, Cheras, Kuala Lumpur, Malaysia

## Abstract

**Introduction:**

Chronic necrotizing pulmonary aspergillosis is an uncommon subacute form of *Aspergillus *infection. It typically occurs in immunocompromised individuals and in those with underlying lung disease. This interesting case highlights the occurrence of this entity of aspergillosis in an immunocompetent middle-aged woman with atypical radiological findings. To the best of our knowledge this is the first case report of chronic necrotizing pulmonary aspergillosis presenting with pleural effusion.

**Case presentation:**

Our patient was a 64-year-old Malay woman with a background history of epilepsy but no other comorbidities. She was a lifelong non-smoker. She presented to our facility with a six-month history of productive cough and three episodes of hemoptysis. An initial chest radiograph showed bilateral pleural effusion with bibasal consolidation. Bronchoscopy revealed a white-coated endobronchial tree and bronchoalveolar lavage culture grew *Aspergillus niger*. A diagnosis of chronic necrotizing pulmonary aspergillosis was made based on the clinical presentation and microbiological results. She responded well to treatment with oral itraconazole.

**Conclusions:**

The radiological findings in chronic necrotizing pulmonary aspergillosis can be very diverse. This case illustrates that this condition can be a rare cause of bilateral pleural effusion.

## Introduction

Chronic necrotizing pulmonary aspergillosis (CNPA) corresponds to an indolent process of lung destruction caused by the Aspergillus fungus, generally A. fumigates [1]. This entity is different from an aspergilloma, as a pre-existing cavity is not needed, although a cavity may develop in the lung as a secondary phenomenon. In contrast to invasive aspergillosis, CNPA occurs over a period of months to years and there is no vascular invasion or dissemination to other organs. The main risk factors are: chronic obstructive pulmonary disease, sequelae of tuberculosis, pulmonary resection, radiation-induced pulmonary fibrosis, pneumoconiosis, cystic fibrosis, pulmonary infarction and sarcoidosis. Other immunosuppression conditions, such as diabetes mellitus, malnutrition, alcoholism, connective tissue diseases and prolonged corticosteroid therapy, are also situations of increased risk [1-3].

Results from chest X-rays may reveal unilateral or bilateral infiltrates with or without cavitation and pleural thickness, especially in the upper lobes and in the upper segments of the lower lobes. In 50% of the cases an aspergilloma occurs simultaneously [1-4]. The definite diagnosis is made through the histological demonstration of tissue invasion by the fungus and the growth of *Aspergillus *species in a culture [2,5].

Other helpful tests include serum IgG antibodies to *Aspergillus *and immediate skin reactivity for *Aspergillus *antigens. Due to the difficulty in confirming the diagnosis, the following diagnosis criteria were established and together are highly indicative of CNPA: characteristic clinical and radiological findings, elevation of inflammatory markers (C-reactive protein (CRP), erythrocyte sedimentation rate (ESR)) and either serological test results positive for *Aspergillus *or the isolation of *Aspergillus *from respiratory samples. Active tuberculosis, non-tuberculosis mycobacteriosis, cavitary histoplasmosis and coccidioidomycosis should be excluded [2,5]. Galactomannan and polymerase chain reaction (PCR) tests from bronchoalveolar lavage, as well as cutaneous sensitivity tests for *Aspergillus*, do not have a confirmed use in diagnosis [2,5].

Therapy with voriconazole or itraconazole has emerged as the first-line treatment and is safer than amphotericin B [6]. The long-term prognosis for patients with CNPA is not well documented. The ideal treatment duration has not yet been defined and depends on the extension of the disease, the patient's response to treatment, the base disease and the patient's immunological condition. In some cases, lifelong therapy may be required [7].

## Case presentation

Our patient, a 64-year-old woman who was a non-smoker with a background history of epilepsy, presented to our respiratory clinic in May 2008 with a six-month history of productive cough with whitish sputum associated with three episodes of hemoptysis. She had no constitutional symptoms. At a primary care clinic a diagnosis of tuberculosis (TB) was considered, but the results of a Mantoux test were negative and findings from the three sputum acid-fast bacilli samples and cultures for TB were also negative. Her symptoms were persistent despite a few courses with oral antibiotics such as oral amoxicillin, azithromycin and moxifloxacin. On examination, our patient was emaciated (body mass index of 19.3 kg/m^2^), hemodynamically stable, apyretic, eupneic and with peripheral oxygen saturation (SpO_2_) of 98% (FiO_2 _21%). She did not have clubbed fingers, palpable cervical lymph nodes or oral thrush.

A bacillus Calmette-Guérin (BCG) scar was present. Her jugular venous pressure was not elevated, and her apex beat was not displaced. An examination of her respiratory system revealed reduced breath sounds at the bases. Based on our patient's history and the physical examination, the differential diagnoses were bronchogenic carcinoma, pulmonary tuberculosis and bronchiectasis.

**Figure 1:**
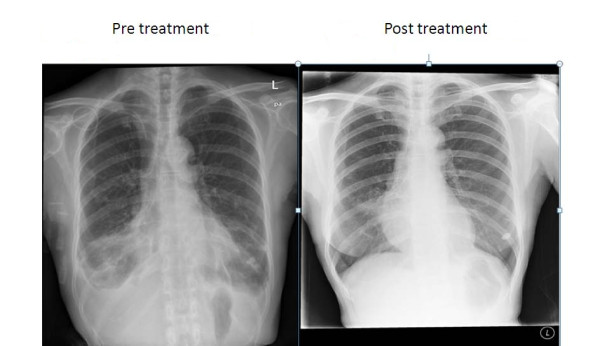
**Pre-treatment and post-treatment chest radiographs**. After two months of antifungal therapy, there was complete resolution of the bilateral pleural effusion with significant improvement in the bibasal consolidation.

The results of initial investigations showed a normocytic normochromic anemia (hemoglobin level of 10.3 g/dL) with normal white cell and platelet counts. Her inflammatory markers were raised: her CRP was 3.24 mmol/L and ESR was 66 mm/hour. Other blood investigations were normal. An initial chest radiograph showed bilateral pleural effusion with bibasal consolidation (Figure 1). A thoracocentesis procedure was not performed as the pleural effusion looked minimal. Subsequently, bronchoscopy was performed, showing an edematous and white coated bronchial tree mucosa; the right lower lobe mucosa had an infiltrate appearance. The mycological bronchoalveolar lavage culture tested positive for *Aspergillus niger*. The mycobacterial cultures tested negative. Unfortunately, a transbronchial biopsy was not performed in the same setting.

Fulfilling the proposed diagnostic criteria by Soubani *et al*. [2], a diagnosis of chronic necrotizing pulmonary aspergillosis was made based on the subacute presentation of six months' duration and microbiological culture that grew *A. niger*. Our patient was discharged with itraconazole 200 mg (syrup) to be taken once daily for an estimated duration of six months. The syrup form was chosen instead of the capsule form for better absorption. After two months of treatment, clinical and radiological improvements were noted on follow-up tests. The itraconazole syrup was continued, aiming for a minimum treatment duration of six months.

## Discussion

The radiological findings of bilateral pleural effusion with lower lung field involvement were not typical of CNPA. Additionally, *A. niger *is a rare causative organism of CNPA, unlike *Aspergillus fumigates*. To the best of our knowledge, this is the first case report of pleural effusion in CNPA. The diagnosis was made six months from the time of onset of symptoms. The atypical presentation delayed the diagnosis. In a case series of 43 patients with CNPA, the upper lobes were frequently involved. The radiological findings in CNPA were very diverse. The reported radiological characteristics were parenchymal consolidation, mycetoma, cavitation, bronchopulmonary fistula, bronchiectasis and emphysema. None of the patients had pleural effusion [2]. However, there were case reports of other forms of aspergillosis, that is, allergic bronchopulmonary aspergillosis and invasive aspergillosis presenting with pleural effusion [4,5].

We believe that the parapneumonic effusion was secondary to pleural inflammation caused by fungal infiltration. The identification of *Aspergillus *in the pleural biopsy would have been confirmatory. This investigation was not performed given that the mycological bronchoalveolar lavage culture tested positive for *A. niger*.

Chronic necrotizing *Aspergillus *pneumonia has a reported mortality rate of 10% to 40%, but the rates could be higher as this entity is under-diagnosed [6]. The wide spectrum and non-specific radiological findings make it more challenging to diagnose this condition.

Our patient was fortunate to have responded well to oral itraconazole. The rapid response was most probably due to her immunocompetent state. In one study, only 38% of patients showed either clinical or radiological improvement with oral itraconazole after more than three months of treatment [2].

## Conclusions

Parapneumonic effusion is a recognized complication of chest infections caused by a wide variety of organisms. This case clearly demonstrates that clinicians should be vigilant and consider CNPA as a differential diagnosis even in healthy patients with unresolving pneumonia manifesting with bilateral pleural effusion.

## Consent

Written informed consent was obtained from the patient for publication of this case report and any accompanying images. A copy of the written consent is available for review by the Editor-in-Chief of this journal.

## Competing interests

The authors declare that they have no competing interests.

## Authors' contributions

SR analyzed and interpreted the data from our patient regarding infectious disease. FMA was a contributor in writing the manuscript. All authors read and approved the final manuscript.
